# Vertebral artery occlusion after anterior cervical discectomy with fusion

**Published:** 2017-10-07

**Authors:** Masatoshi Yunoki, Takahiro Kanda, Kenta Suzuki, Atsuhito Uneda, Koji Hirashita, Kimihiro Yoshino

**Affiliations:** Department of Neurosurgery, Kagawa Rosai Hospital, Kagawa, Japan

**Keywords:** Cervical Vertebrae, Surgery, Vertebral Artery, Arterial Occlusive Diseases, Cerebellar Diseases, Brain Infarction

Anterior cervical discectomy with fusion (ACDF) is an established intervention for cervical degenerative disease. However, on rare occasions, complications, including dural tear, esophageal injury, and dysphagia, may occur.^1^ Here, we report a case of cerebellar infarction due to vertebral artery (VA) occlusion that was diagnosed 2 days after ACDF. 

A 50-year-old woman presented with 3 months history of bilateral arm and neck pain with shoulder radiation and clumsy hand. Cervical magnetic resonance image (MRI) demonstrated cord compression by osteophyte at the level of C5-C6 and C6-C7 (Figure 1, a). She had no medical history of heart valve disease or arrhythmias that could have caused a cerebral embolism. She underwent a 2-level ACDF according to a standard anterior cervical approach. A microscope was used during most of the procedure and the vertebral bodies were separated by the repeated use of a spreader during osteophyte removal. For the interbody spaces of C5-C6 and C6-C7, titanium cages (SynCage-C: Depuy Synthes) with a 7-degree lordotic angle and anterior heights of 7.0 and 5.0 mm were used, respectively (Figure 1, b and c). The patient’s recovery from general anesthesia was normal and her symptoms were alleviated. However, 36 hours after surgery, she complained of mild nausea and vertigo. As these symptoms gradually deteriorated MR angiography and MRI were performed 48 hours postoperatively, which revealed right cerebellar infarction and right VA occlusion (Figure 1, d and e). She responded well to argatroban and was discharged 21 days after surgery without neurological symptoms. During the following 2 years, she has lived a normal life. MR angiography performed 2 years after discharge revealed complete recanalization of the right VA (Figure 1, f and g). 

The VA is particularly susceptible to injury, and VA occlusion following cervical fracture,^2^ chiropractic manipulation,^3^ or even after abrupt head movement^3^ has been reported. A potential mechanism of VA occlusion in such cases is intimal disruption followed by thrombus formation and thus, clot occlusion of the vessel lumen.^3^^,^^4^ The reason why VA occlusion occurred in this case is unknown, however repeated use of a spreader in two consecutive intervertebral space may have triggered this sequence of events. 

**Figure 1:**
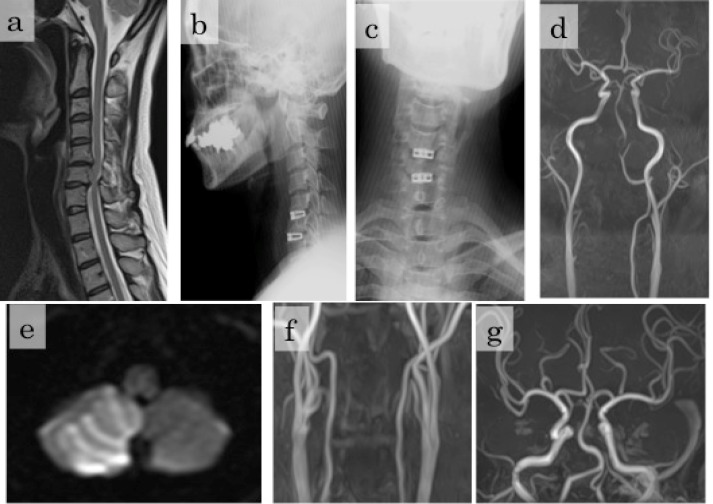
Preoperative cervical MRI (magnetic resonance imaging) demonstrating spondylotic changes at the level of C5-C6 and C6-C7 (a), Lateral and (b), Anteroposterior (A-P) view of immediate postoperative cervical radiography demonstrating titanium cages appropriately placed at C5-C6 and C6-C7 (c), Cervical and cranial MR angiography 2 days after surgery demonstrated right vertebral artery occlusion (d), Diffusion-weighted MRI 2 days after surgery demonstrated cerebellar infarction in the right cerebellar lobe (e), Cervical (f) and Cranial MR angiography 2 two years after discharge revealed complete recanalization of right vertebral artery (g).

Our experience demonstrates the need for surgeons performing ADCF procedures to be aware of this potential complication. There are many other prophylactic treatments of this complication such as maintaining appropriate cervical positioning during surgery or prevention of postoperative dehydration; however, care should be taken to avoid intimal disruption of the VA by overly dilating the intervertebral space.

## Notes:

**How to cite this article** Yunoki M, Kanda T, Suzuki K, Uneda A, Hirashita K, Yoshino K. Vertebral artery occlusion after anterior cervical discectomy with fusion. Iran J Neurol 2017; 16(4): 221-2.
